# Establishing Multi-Dimensional LC-MS Systems for Versatile Workflows to Analyze Therapeutic Antibodies at Different Molecular Levels in Routine Operations

**DOI:** 10.3390/ph18030401

**Published:** 2025-03-12

**Authors:** Katrin Heinrich, Sina Hoelterhoff, Saban Oezipek, Martin Winter, Tobias Rainer, Lucas Hourtoulle, Ingrid Grunert, Tobias Graf, Michael Leiss, Anja Bathke

**Affiliations:** 1 Pharma Technical Development, Roche Diagnostics GmbH, Nonnenwald 2, 82377 Penzberg, Germany; katrin.heinrich@roche.com (K.H.); martin.winter.mw2@roche.com (M.W.); tobias.rainer@roche.com (T.R.); ingrid.grunert@roche.com (I.G.); tobias.graf@roche.com (T.G.); michael.leiss@roche.com (M.L.); 2 Pharma Technical Development, F. Hoffmann-La Roche, Grenzacherstrasse 124, 4070 Basel, Switzerland; sina.hoelterhoff@roche.com (S.H.); saban.oezipek@roche.com (S.O.); lucas.hourtoulle@roche.com (L.H.)

**Keywords:** multi-dimensional liquid chromatography coupled to mass spectrometry (mD-LC-MS(/MS)), (ultra) high-performance liquid chromatography ((U)HPLC), HPLC method characterization, ion exchange chromatography (IEC), immobilized enzyme reactor (IMER), method optimization, post-translational modifications (PTMs), therapeutic antibody

## Abstract

**Background/Objectives:** Multi-dimensional liquid chromatography coupled with mass spectrometry (mD-LC-MS) has emerged as a powerful technique for the in-depth characterization of biopharmaceuticals by assessing chromatographically resolved product variants in a streamlined and semi-automated manner. The study aims to demystify and enhance the accessibility to this powerful but inherently complex technique by detailing a robust and user-friendly instrument platform, allowing analysts to switch seamlessly between intact, subunit, and peptide mapping workflows. **Methods:** Starting from a commercially available Two-Dimensional Liquid Chromatography (2D-LC) system, we introduce specific hardware and software extensions leading to two versatile mD-LC-MS setups, in slightly different configurations. The technique’s efficacy is demonstrated through a case study on a cation exchange chromatography method assessing the charge variants of a bispecific antibody, isolating peak(s) of interest, followed by online sample processing, including reduction and enzymatic digestion, and subsequently mass spectrometry analysis. **Results:** The accuracy and reproducibility of both mD-LC-MS setups proposed in this study were successfully tested. Despite the complex peak patterns in the first dimension, the systems were equally effective in identifying and quantifying the underlying product species. This case study highlights the routine usability of mD-LC-MS technology for the characterization of (ultra) high-performance liquid chromatography (UHPLC) of therapeutic biomolecule. **Conclusions:** The demonstrated reliability and accuracy underscore the practicality of mD-LC-MS for routine use in biopharmaceutical analysis. Our detailed description of the mD-LC-MS systems and insights simplify access to this advanced technology for a broader scientific community, regardless of expertise level, and lower the entry barrier for its use in various research and industrial settings.

## 1. Introduction

Chromatographic approaches are essential for ensuring consistent product quality by evaluating and closely monitoring product variants throughout the product lifecycle of therapeutic antibodies [1,2,3,4]. For a comprehensive understanding, the distinct peaks obtained by these analytical chromatography techniques, such as ion exchange chromatography (IEC), need to be characterized for post-translational modifications (PTMs) using mass spectrometry.

However, PTMs contribute to highly heterogeneous peak patterns when using high resolution (U)HPLC separation methods [5,6]. This is especially important for new antibody formats, which often exhibit a greater number of modifications, resulting in more intricate chromatographic profiles. For these reasons, the offline approach reaches its limits when fully characterizing these new antibody formats. Furthermore, it requires substantial resources in time, material, and labor.

As a powerful alternative for (U)HPLC peak characterization, mD-LC-MS offers major advantages over offline fractionation. The integration of the investigated analytical (U)HPLC method (first dimension) into the mD-LC-MS workflows without the need of any adaptations. This mitigates issues such as loss of chromatographic resolution that can arise from the chromatographic setup during upscaling needed for offline fractionation [7]. The mD-LC-MS facilitates the collection of pure fractions through accurate cuts, which are subsequently transferred and processed automatically before being analyzed by mass spectrometry. In recent years, an increasing number of workflows exploiting this technique have been described, spanning antibody characterization at the peptide, subunit, intact, and native levels [8,9,10,11,12,13,14,15,16,17,18,19,20,21,22,23,24,25,26,27,28,29,30]. The developed mD-LC-MS(/MS) online peptide mapping setups are particularly well suited to fill the gaps that emerge in the peak assignment of complex antibody formats by traditional offline approaches [31]. Besides isolating product variants of interest under representative conditions, saving time and resources, these techniques also minimize the risk of inducing method-related artifacts due to minimal processing steps, short sample hold times, and fast online enzymatic digestion [28]. 

Given the intrinsic complexity of mD-LC-MS systems, especially for online peptide mapping involving several processing steps, the question of stability and reproducibility naturally arises. Several independent studies have consistently demonstrated the reliability and accuracy of this technique, achieving comparable results across different mD-LC systems and offline workflows. Most notably, the inter-laboratory study by Camperi et al. evaluated the performance of three different mD-LC-MS(/MS) setups by characterizing the IEC method of Herceptin^®^ [28]. Reporting the same product variants with comparable relative abundances, underscoring the potential of mD-LC-MS(/MS)-based workflows as a reliable and powerful tool for peak assignment and, hence, in-depth characterization of biopharmaceuticals.

Despite the technical advances in this field over the last years, it is important to note that all relevant studies on mD-LC-MS(/MS) applications have been conducted using custom-built instrumentations by a relatively small number of working groups. While the benefits of this technique are currently widely acknowledged, the lack of commercially available mD-LC systems or publicly accessible blueprints of the configuration of existing systems hinders broader implementation across academic and industrial laboratories. The unavailability of commercial mD-LC-MS systems can be attributed to their complexity, high cost, need for specialized expertise, and insufficient standardization and guidelines.

In this work, we aim to surmount this technical hurdle and improve access to this powerful technique by providing a comprehensive description of our established systems, which are extensions of Agilent’s 2D LC instrument using off-the-shelf modules and valves and can be implemented independently without vendor support. The capabilities of the herein introduced systems are highlighted by a case study involving the characterization of various peaks in an IEC chromatogram at the peptide level.

## 2. Results

As one of the earliest developed systems, Agilent’s 2D-LC paved the way for routine 2D applications in pharmaceutical settings. Although this system has proven highly effective in increasing peak capacity by coupling two orthogonal separation modes or improving MS compatibility, it lacks the required versatility for in-depth characterization of biopharmaceuticals, which involves online processing of the generated cuts. To address this limitation, we devised and refined a specifically tailored mD-LC system that provides the technical means to establish a wide array of analytical workflows.

Starting from the commercially available 2D-LC system [32], the following adaptations were made:

### 2.1. Optimization of the mD-LC System and Method

#### Multiple Heart Cut (MHC) Technology and Active Solvent Modulation (ASM) Valve

MHC technology is crucial for Agilent’s 2D-LC and is based on a 2-position/4-port duo-valve. By diverting the flow from the first-dimension HPLC into two parking decks, each holding six loops with volumes between 10 μL and 180 μL, it enables generating precise and closely spaced cuts, which are successively processed in the following dimensions. By retrofitting our systems, utilizing ASM technology in combination with MHC, problems of incompatibility between the buffers from the first dimension and the employed separation mode in the subsequent dimension can be addressed. The flexibility to adjust the solvent by valve-based dilution enhances the ability to characterize a wide range of 1D methods effectively [33].

### 2.2. Additional Modules

Equipping the 2D-LC system with additional modules increases its versatility. Starting from a single binary pump for the second dimension, we extended the system by introducing three additional pumps, two external 2-position 10-port valves, and two column heaters. In total, a number of three column heaters are employed in the system, all of which are equipped with valves.

To integrate these new modules into the system, we configured a second OpenLab CDS ChemStation software (Rev. C.0.10 [287]) instance, allowing us to host the new modules alongside the original OpenLab CDS ChemStation (Rev. C.0.10 [287]) with integrated Agilent 1290 Infinity 2D-LC software (version A.01.04 [036]), effectively creating a setup akin to a second instrument. More specifically, the first instance hosts the pumps for the 1D and 4D, the UV DAD detector, as well as the MHC valve and the two connected loop decks. The second instance includes all column ovens and valves, as well as the pumps needed for 2D and 3D chromatography. For a detailed list of all modules used, please refer to the Supplementary Materials S1.

### 2.3. Custom Made Plugins

In addition, we implemented two plug-ins in OpenLab CDS ChemStation (Rev. C.0.10 [287]) to enable smooth control of the system. The first, named Valve Event Plug-In, mediates communication between the above-outlined software instances and the MS via contact closure events, utilizing a Universal Interface Box (UIB) board as the corresponding hardware. This facilitates fine-tuning and scheduling of the sequentially executed methods between both instruments and the MS sequence. More specifically, the Valve Event Plug-In triggers the initialization of the instrument methods for both the modules of the second software instance and the hyphenated MS, depending on the switching pattern of the MHC valve and the loop decks.

Details about the functionality are provided in the Supplementary Document S1.

A second plug-in, named “Solvent Selection Valve Switch”, allows the use of all four inlets of a binary pump during a run. Typically, a binary pump allows the selection of only one inlet for each of the A and B channels. These can be selected by default only prior to a run, but not changed during an ongoing analysis. Establishing this option to utilize all available inlets increases the flexibility and reduces the number of pumps required in multi-stage experiments by conferring the possibility to switch between multiple buffers, while still benefiting from the advantages of a binary pump.

### 2.4. Custom-Made Documentation App

The original 2D-LC software, while capable of visualizing the selected cuts in the UV profile and listing their transfer times, lacks functionalities to capture the detailed method and run information of mD-LC experiments. For example, it cannot consolidate data from both software instances or document essential parameters, such as pressure curves from all pumps, which are crucial for process evaluation and troubleshooting. Additionally, it is unable to link the generated MS files to the corresponding cuts, a key requirement for routine operations.

To address these shortcomings, we developed a custom app that enables the generation of comprehensive reports for both method development and routine measurements in a user-friendly manner. The app vastly expands the basic capabilities of the original 2D-LC software. For each processed cut, the comprehensive report encompasses pressure curves for all active pumps and method information of all mD-LC modules (e.g., timetables for pumps, valves and column ovens; and reporting the injection amount). Additionally, the user can attach fluidic flow schemes, information on the utilized columns as well as additional comments, and link the corresponding MS raw data files for each cut. A further feature of this app is the ability to estimate and document the protein amount of the analyte transferred with each cut. This estimation is derived from the total injection amount and the UV area of the cut, providing a quantifiable metric crucial for both method development and routine operations.

Finally, the app allows users to create an editable report with a single click for each injection based on a predefined template, saving time and ensuring that all critical information is systematically extracted and easily accessible, providing a user-friendly solution for documenting and evaluating mD-LC-MS(/MS) experiments.

### 2.5. Selective Workflows

**Figure 1:**
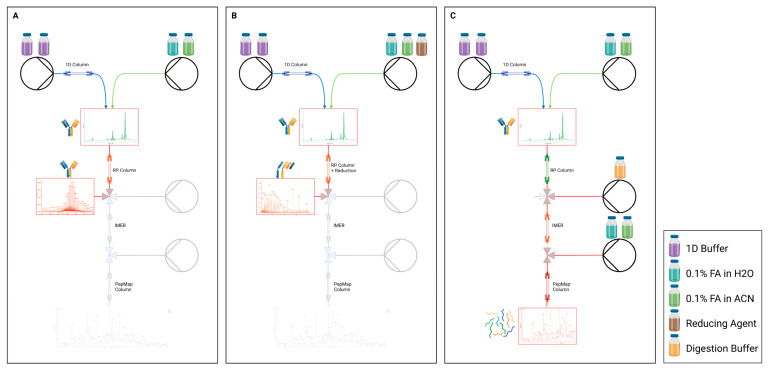
Fluidic flow scheme: Possible selections of different online processing methods and the required flow paths for (**A**) intact, (**B**) reduced and (**C**) peptide mapping through tryptic digestion employing IMERs. (Created in BioRender. Heinrich, K. (2024). https://BioRender.com/x95e115 (accessed on 1 March 2025)).

The employed mD-LC systems allow the analyst to switch between the routine modes of analysis via method selection in the software—(A) intact, (B) reduced, and (B) tryptic peptide mapping (reduced or intact)—without replacing any capillaries or columns. This was achieved through the implementation of two-position valves between the consecutive dimensions. In each of these methods, the valve configurations and flow paths are controlled based on pre-defined timetables (e.g., of pumps, valves and column ovens) to match the corresponding workflow, as shown in Figure 1. Since no hardware adjustments are necessary, the operator saves time, while preventing manual errors and avoiding leaks.

### 2.6. Routine Tryptic Online Peptide Mapping

Besides optimizing the systems in hardware and software, improving the workflows was also a central and ongoing goal. For the online tryptic peptide mapping, a critical step involved the elution of reduced chains of the antibody in the second dimension. To achieve this, the original method employed 60% mobile phase B to elute the antibody chains [15]. Although the concentration of the organic modifier was diluted by the addition of digestion buffer via an implemented T-piece before passing through the IMER, the remaining levels of ACN (11.6%) still impaired the binding conditions for the C18 RP column used in the fourth dimension (4D). As noted by Camperi et al. [28] and Gstoettner et al. [15], this was particularly true for strongly hydrophilic peptides, resulting in no or only partial binding, which in turn affected the ability to quantify these peptides reliably and reduced the overall sequence coverage.

The optimization of the peptide mapping method aimed to increase the binding of hydrophilic and small peptides while ensuring the effective elution of reduced antibodies and complete digestion by the IMER. Developments in the two involved laboratories led to two different strategies, which are briefly described below:

LAB1 Approach: The LAB1 approach involved reducing the ACN concentration required to elute the antibody chains from the short-chain RP (C3) of the second dimension and transfer them to the third dimension IMER. Studies evidenced that a concentration of 35% ACN was adequate for the effective elution of most antibody formats. Additionally, the elution flow rate was decreased and performed at a low flow rate of 50 μL/min to increase the dilution rate with the addition of digestion buffer via a T-piece at 450 μL/min prior to the IMER. This 10-fold dilution resulted in a theoretical remaining value of 3.5% ACN on the C18 column in the fourth dimension during the trapping of the peptides, remarkably enhancing the peptide binding. This approach resulted in a high flow rate of 500 μL/min through the IMER, and the corresponding short contact time of only 12 s proved to be sufficient for the generation of peptides and the performance of valid mass spec analyses. Nevertheless, it is worth noting that to ensure an effective elution, the ACN concentrations had to be varied for different formats. Details about the elution and binding conditions are provided in the Supplementary Document S1.

LAB2 Approach: In LAB2, a more comprehensive approach involved introducing a short C18 pre-column between the IMER and the analytical C18 peptide mapping column (4D), as published by Oezipek et al. [31]. The pre-column reliably acted as a trapping column for the incoming peptides, with the advantage of generating notably less backpressure on the pressure-sensitive tryptic IMER. This allowed for a second dilution by implementing a T-piece downstream of the IMER to dilute the obtained peptides with mobile phase A of the fourth dimension pump (0.1% FA in water) before binding on the C18 column. Consequently, the ACN concentration could be considerably reduced irrespective of the conditions used in the second dimension. The employed dilution factors of 6 before the IMER and additionally 6 after the IMER resulted in a theoretical ACN concentration during peptide binding of 1.6%.

Both configurations from LAB1 and LAB2 were evaluated in the following case study to demonstrate that various adjustments could be made to the methodology while still providing consistent results suitable for routine peak characterization applications.

For detailed information on the settings for the peptide mapping method and optimization strategies regarding small and polar peptides, please refer to the Supplementary Data S1.

### 2.7. Case Study

Utilizing the above-mentioned configurations, we performed a case study across our laboratories to demonstrate that both setups yield consistent results, indicating the effectiveness and reproducibility of our proposed approach. For this, the narrow eluting and low-intensive basic peaks of the IEC method applied to bsAb1 were characterized using online peptide mapping. The identified product species for various peaks were then compared between both systems.

**Figure 2:**
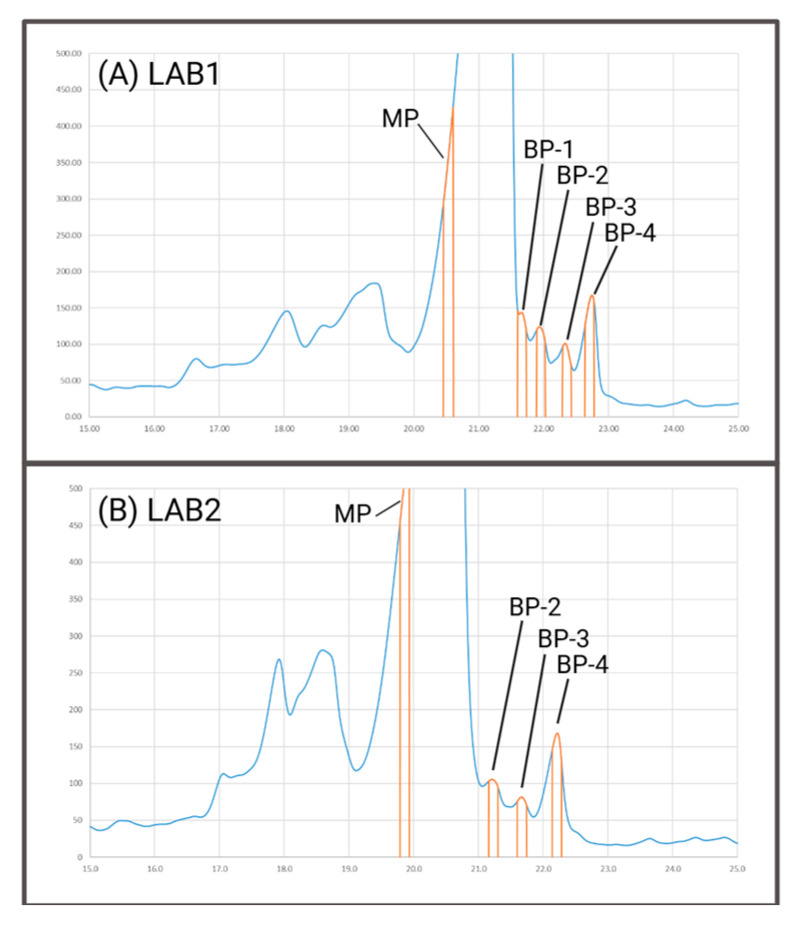
The analyzed cuts (orange) of the first-dimension IEC UV profile (blue) with a 1000 µg injection amount from both laboratories are as follows: (**A**) LAB1 conducted five cutsand (**B**) LAB2 conducted four cuts, all performed in triplicate in three independent IEC runs. Created in BioRender. Heinrich, K. (2025). https://BioRender.com/e48y591 (accessed on 1 March 2025).

In Figure 2A,B representative chromatograms of the IEC method are shown, based on which both laboratories performed cuts in triplicates. These cuts included the main peak (MP) and the most prominent peaks of the basic area (BP-2, BP-3, and BP-4). Upon comparing both chromatograms, an extra peak was apparent in LAB1 at the rear side of the main peak (BP-1), which was additionally assessed. From our experience, this peak is only chromatographically resolved under optimal conditions, which includes factors such as column lot and age. It is important to note that both mD-LC systems utilized the same setup in the first dimension, suggesting that the observed differences in separation performance can be attributed to the respective columns.

First, the quality of the obtained online peptide mapping data was verified by examining the sequence coverage and XIC intensities for peptides. For both laboratories a sequence coverage of 96.9% based on the cut of the MP was achieved. It is important to note that the MP cut used for the determination of the sequence coverage was performed rather at the front side of the main peak than the apex. If the cut were made at the apex, the high injection volume would result in an excessive amount of analyte being transferred into the online workflow, risking an overload of the C18 column (used for the separation of peptides in the fourth dimension).

The second quality parameter was the XIC signal intensity of the wild-type (WT) peptide containing Asp50. Since this residue is prone to modifications (as described below), we decided to evaluate the dataset based on the unmodified peptide. A sufficiently high intensity of this WT peptide is necessary to reliably quantify the amount of the corresponding modified peptides. The XIC intensity of the WT peptide in the MP cut for LAB1 was 4 × 10^7^ (z = 4) and 2 × 10^8^ (z = 5) using an Orbitrap MS, while for LAB2, it was 3 × 10^6^ (z = 3), 8 × 10^6^ (z = 4), and 3 × 10^7^ (z = 5) using a QTof MS. We deemed these values appropriate for an in-depth characterization of the selected peaks.

### 2.8. BP-1: N-Terminal Glutamine (PyroE)—Wildtype

**Figure 3:**
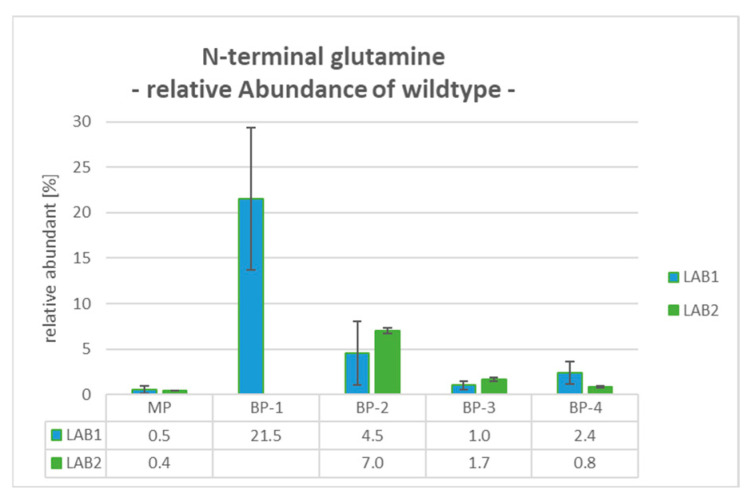
Data of Gln1-peptide WT without pyroglutamate modification (PyroE) gained by mD-LC-MS/MS tryptic peptide mapping: LAB1 determined enriched levels of 21.5% of unmodified Gln1 in BP-1. Average relative abundance [%] with standard deviation bars for triplicates for all fractionated peaks evaluated in LAB1 and LAB2.

For BP-1, which was only observed as a peak shoulder at the rear side of the main peak in LAB1, an incomplete conversion of the PyroE was revealed. The determination is based on the detection of a mass shift of +17 Da relative to the WT peptide caused by the cyclization of the N-terminal glutamine. Compared to the very low levels of Gln1 for the MP (between 0.4% and 0.5%), the relative abundance of Gln1 increased to 21.5% for BP-1 (see Figure 3).

### 2.9. BP-2: C-Terminal Proline Amidation (Pam)

**Figure 4:**
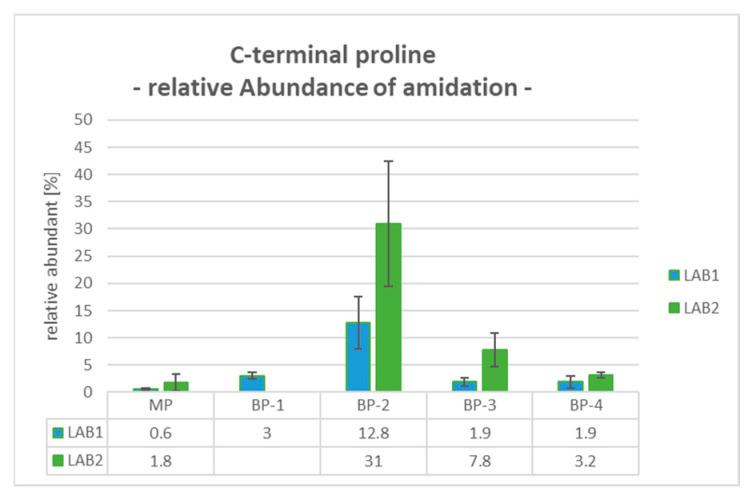
Data of BP-2 for C-terminal Pam modification gained by mD-LC-MS/MS tryptic peptide mapping: Both laboratories reveals high values of Pam in BP-2, whereas the setup of LAB2 gained higher percentages for this modification than LAB1. Average relative abundance [%] with standard deviation bars for triplicates for all fractionated peaks evaluated in LAB1 and LAB2.

The Pam modification of the C-terminus occurs with additional cleavage of the C-terminal glycine-specific peptide and is detectable with a mass shift of −58 Da. Compared to the MP, the levels of C-terminal Pam in BP-2 were significantly increased from 0.6% and 1.8% to 12.8% and 31.0% for LAB1 and LAB2, respectively. For the later eluting basic peaks, the relative proportions were also lower, with 1.9% for LAB1 and levels up to 7.8% for LAB2 (see Figure 4).

### 2.10. BP-3: No PTM Assignment

For BP-3, the online tryptic peptide mapping analysis mD-LC-MS/MS did not detect any considerable changes regarding the levels of PTMs for both systems.

### 2.11. BP-4: Asp50 Succinimide and Isomerization

**Figure 5:**
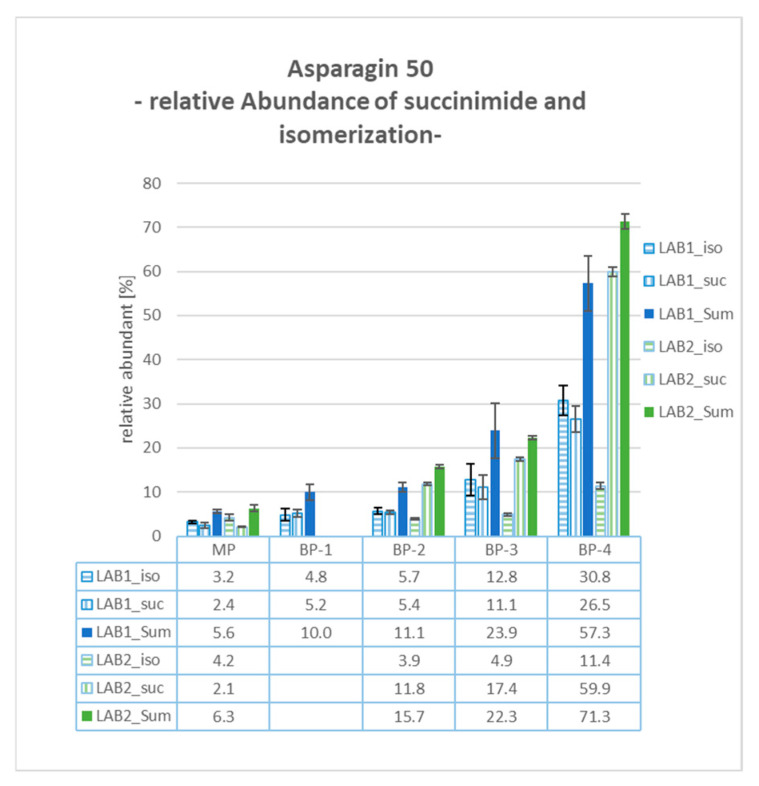
Data of Asp50 succinimide (suc) and isomerization (iso) modification gained by mD-LC-MS/MS tryptic peptide mapping: Summarizing the relative abundance, Asp50 suc and iso indicate high values of modified Asp50 in BP-4 with 57.3% (LAB1) and 71.3% (LAB2). Average relative abundance [%] with standard deviation bars for triplicates for all fractionated peaks evaluated in both laboratories (LAB1 and LAB2).

Both laboratories identified modifications of Asp50 in BP-4, including the formation of succinimide (−18 Da mass shift) and its isomerization. As depicted in Figure 5, the sum of these modifications exhibited relative values of over 50% for both systems, 57.3% in LAB1 and 71.3% in LAB2. These values significantly exceeded those from the other examined cuts, with approximately 6% for the MP and roughly 10% relative abundance for both the basic peaks BP-1 and BP-2. BP-3 also showed an elevated level of Asp50 modifications (23.9% and 22.3% in LAB1 and LAB2, respectively). This is attributed to the non-baseline separation between BP-3 and BP-4, resulting in the partial co-elution of both product species.

## 3. Discussion

mD-LC-MS/MS is recognized as a powerful technique for the analysis and assignment of product species to peaks in (U)HPLC method-based chromatograms. Despite the repeatedly demonstrated potential, the widespread implementation of this technique has been hampered by its inherent complexity. The large number of system components presents a substantial challenge. Not only do the setups have a large footprint, but troubleshooting also becomes more complicated and time-consuming due to the increased potential error sources. Our setups, for example, are physically connected through approximately 40 capillaries and a total of 5 valves (plus MHC valve and loop decks). This complexity necessitates advanced skills for handling and maintenance, with each connection posing a risk for leakages and potentially resulting in extended downtimes.

Method development and optimization for mD-LC-MS/MS can be very time-consuming, as all steps across the different components must be precisely coordinated and temporally aligned to execute workflows effectively. This requires not only a deep understanding of chromatographic techniques and mass spectrometry but also an intimate knowledge of system details (such as dead volumes, contact times for the IMER, and dilution steps). The mD-LC-MS systems utilized in the presented study are based on Agilent’s 2D-LC system, with modifications both on the hardware and software level.

To enhance the systems’ versatility for accomplishing various multi-dimensional chromatographic workflows, such as online peptide mapping, we integrated supplementary pumps, external 2-position 10-port valves, and additional column heaters, all coordinated through a second instance of OpenLab CDS ChemStation software. The installation of custom-made software applications, such as the Valve Event Plug-In and Solvent Selection Valve switch, facilitated seamless communication between software instances and the mass spectrometer, and improved the flexibility of solvent selection during runs. Additionally, a custom documentation app was developed to ensure all run information is systematically organized and to provide the opportunity to generate comprehensive reports, thus streamlining method development and routine analyses.

To markedly decrease downtime caused by issues related to the need for hardware adjustments, we developed methodically optimized flow paths that allow for the switching between routine workflows at the intact, reduced, or peptide level by solely selecting the respective instrument method. This optimization does not only minimize the risk of leakage obstacles, but also contributes to the systems’ ease of use and helps prevent user errors.

To challenge the performance of our proposed setups, we conducted a case study involving online tryptic peptide mapping, which constitutes the most complex workflow of our routine applications. For this, a total of five peaks from an IEC chromatogram of bsAb1 were analyzed with both configurations, which included the main peak (MP) and four very narrow, low-intensity eluting basic peaks (BP-1, BP-2, BP-3, and BP-4).

The high precision of online fractionation in this study is notable, with cuts separated by only 9 s. This precision enables the characterization of nearly co-eluting peaks in complex (U)HPLC UV patterns. It enriches modifications in the transferred cuts, allowing reliable detection and clear assignment of PTMs, despite their low intensity UV levels. This precision arises from two key factors: the direct integration of the HPLC method into the workflow, maintaining resolution and elution profiles, and the MHC technique’s ability to make multiple, precise, and narrow cuts from the HPLC UV profile. These aspects substantially enhance the system’s value and utility compared to conventional offline fractionation.

For the majority of the investigated peaks, same modifications could be clearly assigned to the investigated peaks for both employed workflows, with two exceptions: (1) Only LAB1 was able to fractionate BP-1, which eluted very close to the main peak, and determine it as unmodified N-terminal Gln1. Since both laboratories employ the same configuration for the first dimension, the differences in the 1D performance in resolution could be explained with differences in column lots. (2) For BP-3, no PTM could be identified at the peptide level. This indicates other species, such as mispairings of various chains or conformational changes, which might also lead to charge changes affecting the elution profile. Although these potential product species are not further explored within this study, the versatility of the mD-LC-MS system would allow for detailed examination using non-reduced digestion methods [27], middle up [11], intact [9] or native approaches [12,22,34,35,36,37,38,39] for example.

Considering the quantitative results for the determined product modifications, differences in the relative values were observed between both configurations. For example, in BP-2, the amidation of C-terminal Proline was quantified with a relative abundance of 12.8% for LAB1 and 31.0% for LAB2. Regarding the Asp50 modification in BP-4, LAB2 observed a significantly higher percentage of Asp50 succinimide formation at 59.9%, compared to 26.5% in LAB1. Conversely, the relative abundance of Asp50 isomerization was determined to be 30.8% in LAB1 and only 11.4% in LAB2. These differences can be explained, for example, by the different approaches for reduction and digestion processes both laboratories employed. Asparagine converts to isoAsp through deamidation via a succinimide intermediate, a process that is highly pH-dependent. LAB2 uses TCEP at pH 2.2 and a digestion buffer around pH 6, combined with peptide trapping under low-pH conditions (diluted with 0.1% FA). In contrast, LAB1 uses DTT for reduction, a digestion buffer at pH 8, and no dilution before peptide trapping. The lower pH values in LAB2 favor the stability and accumulation of the succinimide intermediate. In addition, the features of the employed mass spectrometer and the exact position of the cuts can also have an impact on the quantitative results.

Finally, despite the variations in methodologies employed by the two laboratories and the offsets in the qualitatively obtained data, the mD-LC-MS/MS technique consistently demonstrated its reliability in accurately identifying the same qualitative PTMs for these peaks.

## 4. Materials and Methods

bsAb1. The recombinant bispecific IgG1 antibody (bsAb1) is expressed in a Chinese hamster ovary cell system and manufactured at Roche Diagnostics (Penzberg, Germany) using standard cell culture and purification technology. The formulation of drug product material is at a concentration of 120 mg/mL in a His/acetate buffer system (20 mM) at pH 5.5.

Chemicals and Reagents. LAB1 and LAB2 purchased N,N-Bis(2-hydroxyethyl) 2-aminoethanesulfonic acid (BES), N,N-Bis(2-hydroxyethyl) 2-aminoethanesulfonic acid sodium salt (BES sodium salt), calcium chloride anhydrous powder and Tris(hydroxymethyl) aminomethane (TRIS) from MERCK KGaA, Darmstadt, Germany. LAB1 used sodium chloride from MERCK KGaA and 1.4 Dithiothreitol (DTT) from Roche Diagnostics GmbH, Mannheim, Germany. MS-grade formic acid (FA) and 0.1% FA (>99%) in Acetonitrile (ACN) were all purchased from ThermoFisher Scientific™, Rockford, IL, USA. Water was obtained from an arium^®^pro Purification System from Sartorius (Goettingen, Germany). LAB2 used sodium chloride as MS-grade FA from Fluka, St. Louis, MO, USA and ACN (HPLC-grade) as Tris(2-carboxymethyl) phosphine (TCEP) were all purchased from MERCK KGaA. Water was obtained from a Milli-Q Advanced A10 system from MERCK KGaA.

All mobile phases containing salt were filtered through a 0.22 μm polyethersulfone (PES) membrane filter from Corning, Tewksbury, MA, USA in both laboratories.

mD-LC-MS(/MS) System and Peptide Level Workflow. The adjustments and experiments described in this article were conducted at two separate laboratories within the analytical development department at Roche Diagnostics GmbH, Penzberg and Hoffmann-La Roche, Basel, hereinafter called LAB1 and LAB2. Both systems are based on Infinity 2D-LC from Agilent Technology Inc. (Santa Clara, CA, USA) and are extended with additional LC modules from same vendor and are operated using the software OpenLab CDS ChemStation (Rev. C.0.10 [287]) from Agilent Technology.

(1D) IEC and Fractionation: As first dimension, a BioPro IEX-SF column (4.6 × 100 mm, 5 μm) from YMC Co, Kyoto, Japan was employed at a flow rate of 0.8 mL/min and a column temperature of 41 °C to separate bsAb1 charge variants in accordance with the product release method. Mobile phases (A) and (B) consisted of 20 mM BES at pH 6.8 with additional 488 mM sodium chloride for eluent (B). bsAb1 was diluted with (A) to a concentration of 50 mg/mL. An injection amount of 1000 µg of bsAb1 ensures appropriate signal intensity and the loop decks were equipped with 120 µL loops to store cuts for subsequent processing.

The collected material in the loops were processed with subsequent online tryptic peptide mapping. The methods in the two laboratories differ in detail and were as follows: 

(2D) Online RPLC-Reduction: The bsAb1 antibody variants bound on a short chain C3 RP guard column from Agilent Technologies. Mobile phases contained 0.1% FA in water (A) and ACN (B), 20 mM DTT (LAB1) or 20 mM TCEP (LAB2) were employed as reducing agent (C). Following buffer exchange and on column reduction, the reduced bsAb1 samples heavy chain (HC) and light chain (LC) subunits were eluted using multiple step gradients with higher concentrations of mobile phase (B) (35% for LAB1 and 60% for LAB2) at a very low flow of 50 µL/min.

(3D) Tryptic Digestion in Flow-Through Mode: An online digestion was performed in flow-through mode using a Poroszyme™ trypsin immobilized enzyme reactor (IMER) at 37 °C purchased from ThermoFisher Scientific™, Sunnyvale, CA, USA. The digestion buffer (mobile phase A) was 50 mM TRIS with 10 mM calcium chloride at pH 8.0. During digestion, the emerging peptides were trapped on the 4D C18 reverse phase (RP) column peptide mapping column (LAB1) and C18 guard column (LAB2). The flow was diluted with digestion buffer according to the 2D C3 cartridge. After the digestion was finished 50% mobile phase B containing 0.1% FA in ACN were employed to prepare the IMER for the next fraction.

(4D) RP-HPLC Analysis for Peptide Mapping: A Poroshell SB-C18 column (2.1 mm I.D. × 100 mm, 3.5 μm, 130 Å) from Agilent Technology was used as the 4D peptide mapping column. The elution gradient consisted of two linear gradients: 1–40% mobile phase B over 40 min, followed by 40–65% mobile Phase B over 5 min, and then two washing steps at 80% and 90% mobile phase B. Peptide detection was performed using a Q-Exactive HF mass spectrometer (ThermoFisher Scientific™) for LAB1 and an Impact II quadrupole time-of-flight (QTOF) mass spectrometer (Bruker Daltonics, Bremen, Germany) in LAB2.

Custom-made software applications facilitated the execution and are described in the Results Section in more detail: ANGI GmbH (Gesellschaft für angewandte Informatik mbH, Karlsruhe, Germany) provided us the “Valve Event Plug-In” and “Solvent Selection Valve Switch”. Furthermore a documentation and reporting app was developed in co-creation with Leoson BV, Middelburg, The Netherlands.

Supporting Document S1 provides detailed information on the mD-LC-MS(/MS) system and workflows, and on the parameters for data analyzed by means of Protein Metrics software Byologic™ v5.0 (Protein Metrics Inc., San Carlos, CA, USA).

## 5. Conclusions

To date, commercially available LC instruments are restricted to a maximum of two dimensions, and virtually all described mD-LC systems are custom-built, which restricts the broad implementation of multi-step workflows as required for more sophisticated online applications. In this article, we outlined adaptations to the off-the-shelf Agilent 2D-LC system with additional hardware and software co mponents that can be implemented without the support of the manufacturer. This enables beyond-2D-LC applications and provides the flexibility to establish versatile customized workflows. In addition, the thoughtfully designed flow concept of our mD-LC-MS(/MS) systems allows for seamless switching between top-down (intact), middle-up (reduced), and bottom-up (peptide mapping) analyses for routine workflows.

The widespread implementation of these systems for peak characterization of (U)HPLC methods is highly beneficial from our perspective. Compared to offline fractionation, this methodology not only offers the often-cited advantages of resource savings and the prevention of workflow-induced PTMs but also excels in the precision of the performed cuts. This is particularly crucial for new antibody formats, as they frequently display a higher number of modifications, leading to more complex chromatographic profiles.

Two different workflows are outlined to enhance the investigation of small and polar peptides in tryptic online peptide mapping: first, by lowering the elution concentration of the C3 column, and second, by employing a short C18 pre-column for peptide trapping.

The case study highlights the performance of these workflows, demonstrating that both systems reliably assign product variants of bsAb1 to narrow and low-intensity peak patterns in IEC methods. This emphasizes that various adjustments can be made while still obtaining consistent results suitable for routine applications. Ultimately, it showcases the comparability, reproducibility and reliability of our proposed setups, despite its inherent complexity, proving that mD-LC-MS technology is a robust methodology for consistent peak identification.

Looking towards the future, it is crucial to leverage the immense potential of mD-LC-MS and establish it as an industry-wide standard technology. Achieving this would require the development of commercially available mD-LC-MS systems, thereby fostering wider acceptance and adoption among analysts. Additionally, it is essential to build knowledge about mD methods and their benefits within the potential user community.

## Data Availability

Theoriginal contributions presented in this study are included in the article/Supplementary Materials. Further inquiries can be directed to the corresponding author.

## Supplementary Materials

The following supporting information can be downloaded at: https://www.mdpi.com/article/10.3390/ph18030401/s1, Supporting Document S1: Details on Materials and Methods, Principal Functionalities, and Workflow Optimization for ‘Establishing Multi-Dimensional LC-MS Systems for Versatile Workflows to Analyze Therapeutic Antibodies at Different Molecular Levels in Routine Operations’.

## Abbreviations

Two-Dimensional Liquid Chromatography	2D-LC
Acetonitrile	ACN
Active Solvent Modulation	ASM
Basic peak	BP
N,N-Bis(2-hydroxyethyl) 2-aminoethanesulfonic acid	BES
bispecific IgG1 antibody	bsAb1
1.4 Dithiothreitol	DTT
Formic acid	FA
(Ultra) high-performance liquid chromatography	(U)HPLC
Immobilized enzyme reactor	IMER
Ion exchange chromatography	IEC
Isomerization	iso
Mass spectrometer	MS
Multi-dimensional Liquid Chromatography Coupled to a Mass Spectrometer	mD-LC-MS(/MS)
Multiple Heart Cut	MHC
Main peak	MP
C-terminal Proline Amidation	Pam
Post-translational modifications	PTM
Protein Metrics software	pmi
N-terminal Pyroglutamate of Glutamine	PyroE
Reverse phase	RP
Succinimide Modification	suc
Tris(2-carboxymethyl) phosphine	TCEP
and Tris(hydroxymethyl) aminomethane	TRIS
Universal Interface Box	UIB
Wildtype	WT
