# Supplementary material for: Establishing Multi-Dimensional LC-MS Systems for Versatile Workflows to Analyze Therapeutic Antibodies at Different Molecular Levels in Routine Operations
Source: Pharmaceuticals (Basel). 2025 Mar 12;18(3):401. doi: 10.3390/ph18030401 (PMC11944941; doi:10.3390/ph18030401)
Supplement: Supplementary file 1 [file pharmaceuticals-18-00401-s001.zip › pharmaceuticals-3499063-supplementary.pdf]

Supporting Information for:

Establishing Multi-Dimensional LC-MS Systems for Versatile Workflows to Analyze Therapeutic Antibodies at Different Molecular Levels in Routine Operations Katrin Heinrich[1], Saban Oezipek[2], Tobias Graf[1], Sina Hoelterhoff[2], Martin Winter[1], Tobias Rainer[1], Lucas Hourtoulle[2], Ingrid Grunert[1], Michael Leiss[1], Anja Bathke[2*]

[1]Pharma Technical Development, Roche Diagnostics GmbH, Nonnenwald 2, 82377 Penzberg, Germany

[2]Pharma Technical Development, F. Hoffmann-La Roche, Grenzacherstrasse 124, 4070 Basel, Switzerland

*Corresponding author: Dr. Anja BATHKE

*Corresponding author: Dr. Anja BATHKE

mailto:anja.bathke@roche.com

Table of Contents

1. Details to Material and Methods for Multi-dimensional liquid chromatography coupled to a Mass Spectrometer (mD-LC-MS) tryptic Peptide Mapping Workflow 1

1.1 LAB1 1

1.1.1 List of modules used in mD-LC setup 1

1.1.2 Online 4D-LC/MS method (IEC x Reduction-RP-LC x Digestion x Reverse Phase (RP)-HPLC/MS) 1

1.2 LAB2 3

1.2.1 List of modules building mD-LC 3

1.2.2 Online 4D-LC/MS method (IEC x Reduction-RP-LC x Digestion x RP-HPLC/MS) 4

1.3 Data evaluation: Peptide identification and quantification and sequence coverage 4

2 Plug-in UIB board - detailed functionality 5

3 Optimizing Gradient and ACN Concentration for different Antibody Formats in subunit level workflows 6

4 Online peptide mapping – optimization strategies regarding small and polar peptides 7

1. Details to Material and Methods for Multi-dimensional liquid chromatography coupled to a Mass Spectrometer (mD-LC-MS) tryptic Peptide Mapping Workflow

1.1 LAB1

1.1.1 List of modules used in mD-LC setup

Original 2D modules are marked with (A) added modules are marked (B)

Modules hosted under original Two Dimensional Liquid Chromatography (2D-LC) Software are marked with (1) modules under second software license are marked with (2)

Module	Type	Module of 2D-LC (A) / Added Module (B)	Software Host (1) / (2)
Injector	G5668A	A	1
Pump 1 (Ion Exchange Chromatography (IEC))	G5654A	A	1
Pump 2 (Reduction)	G7120A	A	1
Pump 3 (Digestion)	G7111B	B	2
Pump 4 (Desalting)	G7112B	B	2
Pump 5 (Pepmap)	G7104A	B	2
Column compartment 1	G7116B	A	2
Column compartment 2	G7116B	B	2
Column compartment 3	G7116A	B	2
DAD 1	G7115A	A	1
Universal Interface Box (UIB) II	G1390B	B	1
External Valve 1	G1170A	B	2
External Valve 2	G1170A	B	2
MHC Valve & Decks	G1170A	A	1

All capillaries in contact with biomolecules are composed of PEEK or bio-inert material, except for the loops of the Multiple-Heart-Cutting Valve (MHC-Valve), which are made of stainless steel. The valve serves as a transmission path for cuts between the 1D (Ultra) high performance liquid chromatography ((U)HPLC) and 2D dimension and consists of a 1290 Valve Drive (8 port / 2 position) G1170A and two selector valves (deck A and deck B) with six 120 µl loops each. The three column compartments include bio-inert 2 position / 6 port valves. The C18 column is connected via a diverter valve to a ThermoFischer Scientific™ (Sunnyvale, CA, USA) Q-Exactive HF mass spectrometer (MS). The mD-LC setup is controlled by two OpenLab CDS ChemStation instances (Rev. C.0.10 [287]) software from Agilent Technologies (Santa Clara, CA, U.S.A.), while the Q Exactive™ is controlled by Xcalibur™ 3.0.63 software (ThermoFisher™ Scientific).

1.1.2 Online 4D-LC/MS method (IEC x Reduction-RP-LC x Digestion x Reverse Phase (RP)-HPLC/MS)

(^{1}D) IEX Separation and Fractionation:

The charge variant distribution is determined using a YMC BioPro IEX-SF (100 x 4.6 mm, 5 µm) column from YMC (Kyoto, Japan). The column operates at a temperature of 41°C and the flow rate is set to 0.8 mL/min. Injection amount is 1000 µg recombinant bispecific IgG1 antibody (bsAb1). A mobile phase composed of (A) 20 mM BES at pH 6.8, and (B) 20 mM BES, 488 mM sodium chloride at the same pH is used. A linear gradient from 2 % to 15 % (B) is applied, followed by a 5-minute washing step at 100 % (B) before the composition returns to 2 % (B). A DAD Detector records UV280 nm for 1D.

As the parameters chosen for the 1D analysis are taken from the release method of bsAb1, all parameters have been successfully validated and were not optimized for the investigation shown here. An exception is made for the injection amount, which is increased to 1000 µg to ensure sufficient analyte transfer for the less intensive basic peaks.

Fractions of 120 µL volumes are obtained from the IEX profile using predefined cutting times in the OpenLab CDS ChemStation 2D-LC Method configuration software. This process is based on a 1D UV280 nm chromatogram and employs multiple heart cuts mode.

(^{2}D) Online RPLC-Reduction:

Subsequently the collected cuts are flushed out of the loops. A Valve Event Plugin programmed by ANGI GmbH (Software Engineering und Laborinformatik, Karlsruhe Germany) gives the Contact closure to second OpenLab CDS ChemStation panel automatically when the MHC-Valve or the valves of the loopdecks switches to transfer the collected cut onto the column of ^{2}D. (See chapter 0 below)

The processed fraction is transferred onto a ZORBAX Stable Bond 300 C3 (4.6 x 12.5 mm, 5 µm) guard cartridge from Agilent Technologies. The column temperature is fixed at 80°C. Mobile phases A and B contain 0.1 % FA in water (A) and Acetonitrile (ACN) (B). Mobile phase C, containing 20 mM DTT, is used to reduce bsAb1 samples into heavy chain (HC) and light chain (LC) subunits. After a short flushing step with 1 % mobile phase B, the reduced bsAb1 subunits elute over the next 50 minutes with multiple gradients up to 35 % mobile Phase B. During the elution of the subunits, the valve located between the 2D and 3D columns switches, brings C3 cartridge and immobilized enzyme reactor (IMER) in-line enabling the transfer of the antibody's subunits at low flow of 50 µl/min to the third dimension trypsin IMER. After the transfer, the valve switches back, and the C3 cartridge is flushed with three step gradients up to 90 % mobile phase B for cleaning purposes.

(^{3}D) Tryptic digestion in flow-through mode:

A Poroszyme™ immobilized trypsin cartridge (2.1 mm x 30 mm) supplied by ThermoFisher Scientific (Sunnyvale, CA, USA) is employed. The column temperature is set to 37°C. The digestion buffer (mobile phase A) is composed of 50 mM TRIS and 10 mM calcium chloride, pH 8.0. Mobile phase B contains 0.1 % FA in ACN. While receiving bsAb1 subunits from C3, the IMER cartridge is also in line with a 4D peptide-mapping column. As a result, the peptides produced by the trypsin cartridge are trapped onto the 4D column. With implementing a tee piece before the IMER the 50 µl/min flow from 2D cartridge is diluted with 450 µl/min digestion buffer, resulting in 14 seconds residence time for the bsAb1 subunits on the trypsin cartridge. When the digestion and the transfer of the obtained peptides is finished the valve between the IMER and C18 column switches to disconnect the IMER and the 4D C18 RP, and the IMER is flushed with 50 % mobile phase before re-equilibrated with 99 % mobile phase A.

(^{4}D) RP-HPLC analysis for peptide mapping:

The RP-LC Poroshell SB-C18 column (2.1 mm I.D. x 100 mm, 3.5 µm, 130 Å) used for the peptide mapping analysis is supplied by Agilent (Waldbronn, Germany). Mobile phases A and B contain 0.1 %

FA in water (A) and ACN (B), respectively. The flow rate is set to 0.3 mL/min. The column temperature is set at 60°C.

At the beginning, the 4D column is washed with 90% B and equilibrated with 1 % B. When the digestion on the trypsin column takes place, the connecting valve between 3D and 4D switches in line and the resulting peptides are transferred and trapped onto the peptide-mapping column for 54 minutes. After that, the valve switches back and the 4D column is flushed with 1% B for the next 3 minutes. Subsequently, the trapped peptides are eluted with the following gradient conditions: 1–40 % B in 40 minutes, 40-65 % mobile phase B in 5 minutes, followed by two 2-minute washing steps at a concentration of 80 % and 90 % mobile phase B, and transferred to a Q-Exactive HF MS at a flow rate of 0.3 mL/min.

MS parameters:

The HESI parameters are set as follows: Spray voltage: 3.5 kV, capillary temperature: 256°C, Aux. gas temperature: 350°C, in-source CID: 0 eV and S-Lens RF level: 30.0 eV. Sheath gas, auxiliary gas, sweep gas flow rates are set to 48, 11 and 2 units. For the MS method: Full MS in positive mode; scan range: 200-2000 m/z, 1 spectrum/s, Resolution: 60’000, AGC target: 1e6 and maximum injection time: 150 ms; dd-MS2 experiments, Resolution: 15000, AGC target: 1e5, maximum injection time: 100 ms, loop count: 10, isolation window: 2.2 m/z, NCE: 26, 28, 30.

1.2 LAB2

1.2.1 List of modules building mD-LC

Original 2D modules are marked with (A) added modules are marked (B)

Modules hosted under original 2D-LC Software are marked with (1) modules under second software license are marked with (2)

Module	Type	Module of 2D-LC (A) / Added Module (B)	Software Host (1) / (2)
Injector	G5667A	B	1
Pump 1 (IEC)	G4220A	B	1
Pump 2 (Reduction)	G4220A	B	2
Pump 3 (Digestion)	G5611A	B	2
Pump 4 (Desalting)	G1311B	B	2
Pump 5 (Pepmap)	G4220A	B	1
Column compartment 1	G7116B	B	2
Column compartment 2	G7116B	B	2
DAD 1	G1314F	A	1
Internal Valve 1	G1170A	A	1
Internal Valve 1	G1170A	A	1
External Valve 3	G1170A	A	1

MHC-Kit	G4236A	A	1

1.2.2 Online 4D-LC/MS method (IEC x Reduction-RP-LC x Digestion x RP-HPLC/MS)

(^{1}D) IEX Separation and Fractionation:

The IEX Separation is performed identical to LAB1, 1.1.2 ((1D) IEX Separation and Fractionation).

(^{2}D) Online RP-LC-Reduction:

The reduction is performed identically to LAB1 Supp. Chapter 1.1.2 ((2D) Online RP-LC-Reduction), with the following modifications: The processed fraction is transferred onto a Poroshell Stable Bond 300 C3 (2.1 x 12.5 mm, 5 µm) guard cartridge from Agilent Technologies. The column temperature is set to 70°C instead of 80°C, the reduction time is set to 5 minutes, three gradient steps are used up to a maximum of 60 % mobile phase B, and the total elution time is 25 minutes instead of 50 minutes.

(^{3}D) Tryptic digestion in flow-through mode:

The digestion is performed identical to LAB1 Supp. Chapter 1.1.2 ((3D) Tryptic digestion in flow-through mode), with the following modifications: The temperature is set to 38°C instead of 37°C.

(^{4}D) Trapping and desalting:

In order to reduce the ACN concentration while trapping, the peptides are diluted by adding 1.5 ml/min Milli-Q water with 1 % ACN via a T-piece. To protect the trypsin IMER by the increased backpressure at high flow rates a pre column Acquity® UPLC BEH C18 RP column, 130Å, 1.7 µm, 2.1 mm X 5.0 mm by Waters cooperation inc. is used as a trapping column.

(^{5}D) RP-HPLC analysis for peptide mapping:

The peptide mapping is performed identical to LAB1 Supp. Chapter 1.1.2 ((4D) RP-HPLC analysis for peptide mapping), with the following modifications: The column temperature is set to 40°C and the Acquity® UPLC BEH C18 RP column, 130 Å, 1.7 µm, 2.1 mm X 150 mm by Waters cooperation inc. is used. Peptide detection was performed using an Impact II quadrupole time-of-flight (QTOF) mass spectrometer (Bruker Daltonics, Bremen, Germany).

MS parameters:

The ESI parameters are set as follows: spray voltage 4.5 kV, end plate offset 500 V, nebulizer 2.0 bar, dry gas flow 11.0 L/min and dry temperature 220°C. For the MS method: MS mode positive, scan range 150-2000 m/z, spectra rate 2.00 Hz, fragmentation Collision-induced dissociation (CID) and the MS/MS auto cycle time is 3.0 seconds.

1.3 Data evaluation: Peptide identification and quantification and sequence coverage

We use PMI-Byologic™ v5.0 (Protein Metrics Inc, San Carlos, CA) to evaluate the generated data from both laboratories. We employ a list of in-silico peptides to identify modifications and determine their relative percentages. For determining sequence coverage and detailed peptide identifications, we utilize the PMI-Byologic™ search of MS/MS data.

In-silico Options

Missed Cleavages Max: 0
Digestion: Trypsin @ R, K | C-term
Digestion specify: Fully specific
Peptide Minimum Mass: 300
Peptide Maximum Mass: 9000
Enable in-silico digest: true
Disulfide mode: Free peptides only

MS/MS Search Parameters

Minimum score (MS2 search): 15
Maximum precursor m/z error (± ppm): 25
Peptide termini: Fully specific
Maximum number of missed cleavages: 1
Precursor tolerance: 5.0 ppm
Fragment tolerance: frag: cid 0.5 Da
Charges applied to charge-unassigned spectra: 1, 2, 3
Precursor mass max: 10000

Included Modifications

Deamidated / +0.984016 @ N
Glu->pyro-Glu / -18.010565 @ NTerm E
Gln->pyro-Glu / -17.026549 @ NTerm Q
Oxidation / +15.994915 @ M, W
Hex / +162.052824 @ K
nsuc / -17.026549 @ N
dsuc / -18.010565 @ D
Lys-loss / -128.094963 @ Protein CTerm K
Prolinamide / -186.22 @ Protein CTerm
OxidationW / +3.99492 @ W
K-Hydroxylation / +15.994915 @ K
P-Hydroxylation / +15.994915 @ P

2 Plug-in UIB board - detailed functionality

At the start of an analysis, the sequence tables for both the first and second instruments, as well as the sequence for the mass spectrometer (MS), are initiated. The 1D method is executed on the first instrument, as well as the cuts and the 4D method are configured in the 2D-LC method editor within the first instrument. The sequence tables of the second LC instrument, along with the MS method, encompass the required parameters for the processing of the fragment and MS analysis. At the onset of the workflow, only the modules of the first instrument are in operational mode, whereas the modules in the second OpenLab CDS ChemStation instance and the MS remain in standby mode, remaining inactive during the collection of 1D peaks/cuts. Upon transferring a cut from one of the loop decks to the next dimension, a contact signal is triggered via the UIB board using the Valve Event Plugin. This activates both the modules in the second instance and the sequence of the MS.

This synchronization aligns all cycle times between (I) the 2D-HPLC methods on the first instrument, (II) the methods/timetables of the second instrument, and (III) the MS. The second instrument and the MS method, which have a shorter runtime than the transfer time of the first instrument, transition to standby mode after processing a cut, ready to receive the start signal for the next cut and proceed with the next line of the sequence upon the transfer of the subsequent cut.

Depending on the investigation's requirements, the processing method of the second instrument may vary between cuts within a run or remain identical. Utilizing the Valve Event Plugin, it is unnecessary to calculate switching and delay times for processing each cut.

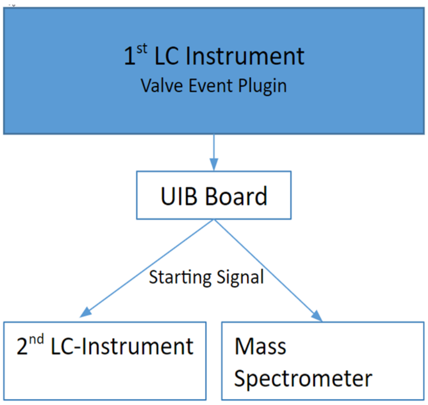

Figure 1, The Valve Event Plug-in triggers the UIB Board to send starting signals for each cut to the modules of the 2nd instrument and MS.

3 Optimizing Gradient and ACN Concentration for different Antibody Formats in subunit level workflows

The comparison of different C4 RP columns from established manufacturers showed that both the Biozen Intact from Phenomenex (2.1 x 150 mm, 3.6 µm) and Acquity UPLC BEH from Waters (2.1 x 50 mm, 1.7 µm) resulted in excellent separation of reduced antibodies, thus serving as standard columns for reduced measurements in our laboratories.

The investigation of therapeutic antibodies at the heavy and light chain subunit levels is frequently employed due to its ability to precisely analyze events such as fragmentation and clipping. Prior to this study, gradients with high ACN concentrations (e.g., 60 %) were utilized to ensure complete elution of the reduced chains. However, the steep isocratic gradients often resulted in the co-elution of antibody chains, which is undesirable as it may mask modifications during mass spectrometric analysis.

We aimed to identify an appropriate gradient and ACN concentration that allow for:

1. The elution of all subunits from various antibody formats,
2. Keeping the gradient as shallow as possible by minimizing the maximum ACN concentration,
3. Maintaining a short method time to ensure practicality in reduced online workflows.

To achieve this, we reduced molecules on-column and examined their UV-280 nm elution profiles, as shown in Figure 2.

The gradient demonstrated distinct elution profiles for the therapeutic antibodies under investigation. Almost all formats achieved complete elution at 35 % ACN, except for mAb2 and mAb3, which include interleukin linked to the Fc region of the antibodies. These two formats exhibited peaks during the 100 % ACN wash step (Figure 2).

Ultimately, the refined gradient starts at 28% and peaks at 35% ACN, which is appropriate for most of the tested antibody formats. Despite the narrow ACN range, the implemented method successfully eluted the individual chains separately, thus improving the detection of modifications. For antibody formats containing Fc-linked interleukin, adjustments to higher ACN concentrations may be necessary to ensure complete elution.

This study reduced the gradient to just 4 minutes, expediting the online workflow for reduced-level analysis.

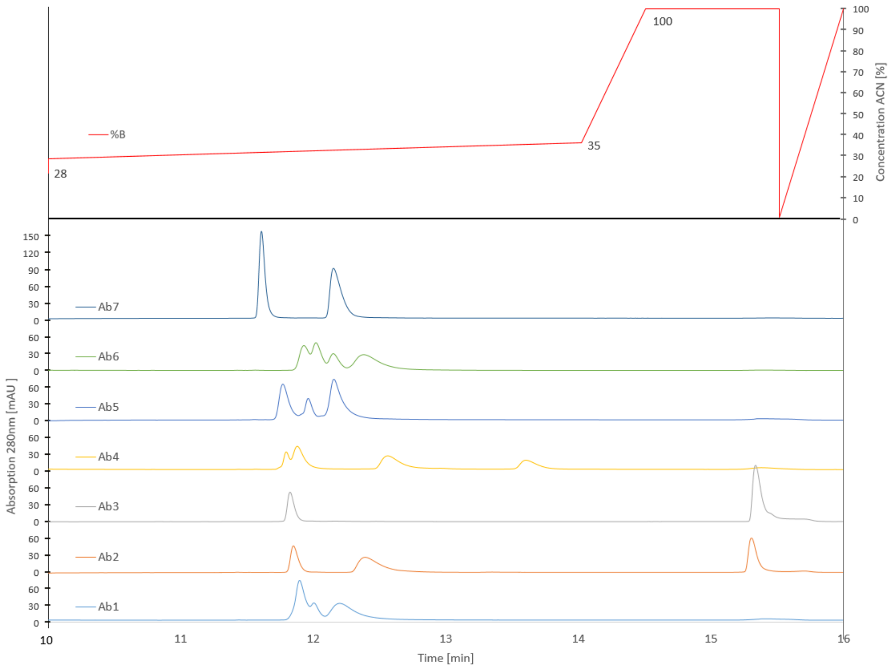

Figure 2, RP-C4 UV 280 mAU elution profiles of different antibody formats, including bispecific, 2+1 formats, Fc linked; employed column: Acquity UPLC BEH from Waters (2.1 x 50 mm, 1.7 µm)

4 Online peptide mapping – optimization strategies regarding small and polar peptides

The tryptic online peptide mapping method was optimized to ensure efficient digestion using trypsin IMERs, while also enhance the binding of the generated tryptic peptides on the subsequent C18 RP column. There are many parameters that have an impact on the binding of the generated peptides, such as column type and column temperature. However, the optimization specifically focused on reducing the ACN concentration during peptide trapping without negatively affecting the digestion efficiency of the upstream trypsin IMERs. The prevailing ACN concentration during peptide binding was found to be a critical aspect. The following factors have the most influence on the ACN concentration in the mD-LC-MS peptide mapping workflow: (1) ACN content for elution of the reduced chains from the short-chain RP, (2) dilution ratio with ACN-free buffers through T-pieces, and (3) flow rates applied. Whereas all this factors also influence the digestion efficacy of trypsin IMER.

LAB1 in Camperi et al [28] integrated the findings described in Chapter 2 for reduced analyses into its peptide mapping workflow. To lower the ACN content for elution, the concentration was reduced from 60 % ACN to 35 % ACN compared to the reference method and in accordance with the data shown in chapter 3. Furthermore, flow rates were adjusted at various points in LAB1, increasing the dilution factor before the trypsin IMER to 10. These changes result in a calculated ACN concentration of 3.5 % for peptide trapping, down from the original 11.6 %. Details can be found in Table 1. **Oezipek et. al.** [31] recommend a higher concentration of 50 % ACN to ensure complete elution of reduced mAb from

the second dimension RP column. To address this relatively high ACN concentration, further dilution is necessary. LAB2 achieved this using a C18 pre-column to trap the peptides after their generation. This very short column, due to its low backpressure, allowed for a significant dilution step between the trypsin IMER and the C18 trap column via a T-piece. Consequently, the ACN concentration during trapping was reduced to a very low 1.2 %. Details can be found in Table 1. It is also noteworthy that both laboratories switched to short-chain C3 cartridges in the second dimension. Due to the small volume of these cartridges, the cycle times for desalting, reduction, and elution can be significantly reduced. Additionally, the C3 cartridges usually required a lower ACN concentration for the complete elution of the trapped mAb.

Overall, the optimization of the method boosted the sequence coverage by about 7 to 8 %, resulting in final coverages of 96 % and 97 % for Trastuzumab in LAB1 [28] and for Oezipek et al [31], respectively. Based on our experience, we emphasize that each mD system is unique, utilizing different modules and varying in dead volumes. Therefore, method optimization and fine-tuning of critical parameters must be performed independently for each instrument (see Table 1).

Table 1,: Online peptide mapping - optimized workflow parameters (DF: Dilution Factor to reduce ACN via T-piece (after C3 RP with digestion buffer and before C18 trapping with waters containing 0.1 % formic acid); DT: digesting time, theoretical duration time of reduced mAb with set flow; ACN: maximal concentration of ACN on RP columns for elution on short chain reduction column and trapping on C18, value for peptide mapping column results from dilution factors and is theoretical; CV: column volume)

	Gstoettner et al. [15]	LAB1 of Camperi et al. [28]	Oezipek et al. [31]
**Reducing on short chain RP columns**			
column	AdvancedBio RP cartridge C4 (2.1×12.5 mm)	ZORBAX StableBond 300 C3 (4.6 x 12.5 mm, 5 µm)	Poroshell StableBond 300 C3 (2.1 x 12.5 mm, 5 µm)
flow [µl/min]	60	50	50
ACN [%]	60	35	50
**Digestion**			
flow [µl/min] / DF	250 / 5.2	450 / 10.0	250 / 6.0
trypsin IMER	STYROSZYME® (2.1x100 mm, 346 µL CV)	Poroszyme™ (2.1x30 mm, 104 µL CV)	Perfinity® (2.1x100 mm, 346 µL CV)
DT [sec]	67	14	75
**Trapping of Peptides**			
flow [µl/min] / DF	no	No	1700 / 6.7
column	no	No	InfinityLab Poroshell 120 SB-C18 (3.0 x 5 mm, 1.9/2.7 µm)
**Peptide Mapping**			
ACN [%]	11.6	3.5	1.2
column	UPLC ACQUITY BEH C18 (2.1 × 50 mm, 1.7 µm)	RPLC XSelect™ CSH C18 (2.1 x 100 mm, 3.5 µm)	InfinityLab Poroshell120 SB-C18 (2.1 x 150 mm, 1.9 µm)
sequence coverage	LC 94 %, HC 86 % (appr. overall 89%)	96 %	97 %
